# Modulation of Immune Responses by Platelet-Derived ADAM10

**DOI:** 10.3389/fimmu.2020.00044

**Published:** 2020-02-05

**Authors:** Stefanie Maurer, Hans-Georg Kopp, Helmut R. Salih, Korbinian N. Kropp

**Affiliations:** ^1^Clinical Collaboration Unit Translational Immunology, German Cancer Consortium (DKTK), Department of Internal Medicine, University Hospital Tuebingen, Tuebingen, Germany; ^2^DFG Cluster of Excellence 2180 ‘Image-guided and Functional Instructed Tumor Therapy’ (IFIT), University of Tuebingen, Tubingen, Germany; ^3^Department of Radiology, Memorial Sloan Kettering Cancer Center, New York, NY, United States; ^4^Departments of Molecular Oncology and Thoracic Oncology, Robert-Bosch-Hospital Stuttgart, Stuttgart, Germany; ^5^Department of Hematology, Medical Oncology and Pneumology, University Medical Center of Mainz, Mainz, Germany

**Keywords:** platelets, tumor, immune evasion, ADAM10, ectodomain shedding

## Abstract

Platelets have a crucial function in maintaining hemostasis. However, beyond their role in coagulation and thrombus formation, platelets have been implicated to affect various pathophysiological conditions such as infectious diseases, autoimmune disorders, and cancer. It is well-established that platelets aid local cancer growth by providing growth factors or contributing to cancer angiogenesis. In addition, they promote metastasis, among others by facilitation of tumor cell-extravasation and epithelial-to-mesenchymal-like transition as well as protecting metastasizing cancer cells from immunosurveillance. A variety of membrane-bound and soluble platelet-derived factors are involved in these processes, and many aspects of platelet biology in both health and disease are regulated by platelet-associated metalloproteinases and their inhibitors. Platelets synthesize (i) members of the matrix metalloproteinase (MMP) family and also inhibitors of MMPs such as members of the “tissue inhibitor of metalloproteinases” (TIMP) family as well as (ii) members of the “a disintegrin and metalloproteinase” (ADAM) family including ADAM10. Notably, platelet-associated metalloproteinase activity not only influences functions of platelets themselves: platelets can also induce expression and/or release of metalloproteinases e.g., in leukocytes or cancer cells, and ADAMs are emerging as important components by which platelets directly affect other cell types and function. This review outlines the function of metalloproteinases in platelet biology with a focus on ADAM10 and discusses the role of platelet-derived metalloproteinases in the interaction of platelets with components of the immune system and/or cancer cells.

## Introduction

The main function of platelets in the healthy individual is maintenance of hemostasis, i.e., prevention of blood loss and protection of vascular integrity. However, the presence of platelets can, under pathophysiological circumstances, be also unfavorable. Platelets are the main culprits during arterial thrombosis causing tissue ischemia with widespread consequences for the affected individual. Moreover, the presence of platelets is also exploited by cancers to aid both their local progression as well as formation of metastasis. Cancer cells entering the blood stream are rapidly surrounded/coated by platelets, leading to enhanced survival of circulating tumor cells and facilitation of metastasis [reviewed in (1, 2)]. The underlying mechanisms are multifaceted and comprise, among others, supply of growth factors, contribution to endothelial adhesion, and mediation of an epithelial-to-mesenchymal-like transition (EMT-like) (3). This is exemplified by studies in mice where metastasis-formation is inhibited in the absence of platelets (4, 5). Additional depletion of natural killer (NK) cells reverted the anti-metastatic effect of thrombocytopenia, and it was assumed that coating platelets provide mechanical shielding from NK cell attack (6). However, recent evidence revealed the involvement of more complex mechanisms such as conferring of a pseudo-self-phenotype (7, 8). A variety of platelet-derived molecules are implicated in these observations. Among them are members of the metalloproteinase (MP) family such as matrix metalloproteinases (MMPs). While the role of MMPs in platelets in the context of cancer and immunity has been reviewed elsewhere (9), platelets also express other MP family-members such as “a disintegrin and metalloproteinase” (ADAM)s. Given the important role of ADAMs in immunity (10) and cancer (11), the present review highlights the relevance of platelet-derived ADAMs, in particular ADAM10 (herein referred to as pADAMs and pADAM10, respectively) for the role of platelets in cancer and tumor immunology.

## ADAMs in Platelets

In humans, 24 *ADAM* genes including four pseudogenes have been described which give rise to 20 Type-I transmembrane proteins representing the functional members of the ADAM family (excluding the ADAM-like soluble ADAMDEC-1 protease) (12–14). They share a common structure comprising a signal peptide at the N-terminus followed by a prodomain, the (catalytically active) metalloproteinase domain, a disintegrin and a cysteine-rich domain, an EGF-like domain (except in ADAM10 and ADAM17), the transmembrane domain and a cytosplasmic tail at the C-terminus (12). Of note, only 12 ADAM-family members bear a catalytically active site in their metalloproteinase domain, suggesting that ADAM proteins may also exert non-proteolytic functions such as modulating cell-cell interaction through their integrin-binding disintegrin domain (12, 13). Of the proteolytically active ADAMs, ADAM10, and ADAM17 have most extensively been investigated (12) ADAM10 and ADAM17 are vital in a plethora of biological processes, and mice carrying a classical knockout of either protease die during embryonic development (15, 16). Information about the presence of ADAM-family members in platelets is derived from analyses of the platelet-proteome. Studies of the complete platelet-proteome by several investigators point to the presence of pADAM9 and pADAM10 (17, 18), whereas other groups did not detect any ADAM-family members (19, 20). Platelet membrane enrichment techniques ultimately confirmed the presence of pADAM9 and pADAM10 and additionally lead to the identification of pADAM17 (21, 22). Notably, pADAM10 is among the 40 most abundant membrane-associated proteins at ~2,000–4,000 molecules/platelet (22). In addition, RNA-sequencing data revealed that platelets also seem to carry significant amounts of *ADAM9* and *ADAM10* transcripts, which may serve as an additional pool for translation e.g., upon platelet-activation (23). These data are corroborated by mechanistic studies describing substrates of pADAM10 and pADAM17 in platelets and confirming the expression of proteolytically active proteases. Mice with a platelet-specific knockout of ADAM10 and/or an ADAM17 knockout confined to the hematopoietic system have been generated (24). These mice are viable, have normal platelet counts and represent an ideal model to investigate the role of pADAMs under physiological and pathophysiological circumstances. The function of pADAM9 in platelets, however, is less well-established. Classical ADAM9-knockout mice display no apparent phenotypical changes compared to wildtype mice, arguing against a major role of ADAM9 under physiological circumstances (25). One *in-vitro* study suggests that the disintegrin-domain of pADAM9 may interact with tumor-cell expressed integrins such as αVβ3, thereby mediating the recruitment of tumor cell-platelet conjugates to collagen (26). Collagen induced platelet activation itself was not dependent on ADAM9 (26). Along this line, studies of the ADAM9-downregulating micro-RNA miR-126 in CD34^+^ derived platelet-like structures and in the megakaryoblastic cell line MEG-01 confirmed ADAM9-mRNA expression, but also did not identify a major role of pADAM9 in platelet activation (27, 28). This is, as already stated above, in stark contrast to pADAM10 and pADAM17, which play major roles in platelet biology.

## Function of (p)ADAM10

For ADAM10, which is at the center of this review, over 40 different substrates have been described (29, 30). Many of these are expressed on platelets. A comprehensive mass spectrometry-based analysis of proteins in the supernatant of activated platelets identified over 1,000 proteins, of which 69 are membrane anchored and therefore potentially accessible to proteolytic shedding by pADAM10 (31). Of these 69 proteins, shedding of 7 substrates was abrogated by ADAM17 inhibition (31), leaving 62 proteins to be potentially shed by pADAM10. We performed a PubMed literature search for each of these 62 platelet-expressed proteins to select for confirmed substrates of ADAM10. In order to obtain an unbiased list of potential substrates we then started with confirmed ADAM10 substrates (29, 30, 32–35) and individual publications and performed a second PubMed literature search to select for those proteins which are reportedly expressed on platelets. Of note, we here summarize studies deriving from different experimental strategies without applying restrictions e.g., a certain abundance in mass spec studies. Using this approach, we identified a total of 35 putative and 3 confirmed substrates of pADAM10 on platelets (Table 1). The pADAM10 sheddome may still be larger and contain proteins that could not be detected by the mass spectrometry-based techniques employed. Future studies however will be indispensable to validate whether and to which extent these putative substrates are indeed cleaved by pADAM10 as compared to ADAM10 from other sources or different sheddases.

**Table 1:** Platelet-expressed ADAM10 substrates.

**Substrate**	**Gene name**	**Expression on platelets**	**ADAM10 substrate**
Amyloid beta precursor protein	APP	(36, 37)	(38, 39)
Amyloid like protein 2	APLP2	(31, 40)	(41)
Axl	AXL	(42, 43)	(44)
BRI2	ITM2B	(17)	(45)
Cadherin 10	CDH10	(46)	(34)
Cadherin 6	CDH6	(46)	(34)
CD147	BSG	(47, 48)	(49)
CD23	FCER2	(50, 51)	(52)
CD40L	CD40LG	(31)	(53, 54)
CD44	CD44	(17, 55)	(56)
cMet	MET	(57)	(58)
Collagen alpha-1(XI) chain	COL11A1	(59)	(34)
CXCL16	CXCL16	(60)	(61)
Desmoglein-2	DSG2	(62)	(63)
E-cadherin	CDH1	(31, 64, 65)	(66)
Epidermal Growth Factor	EGF	(31, 67, 68)	(69)
FasL	FASLG	(70, 71)	(72)
GPV^*^	GP5	(31)	(73)
GPVI^*^	GP6	(31)	(73, 74)
H(+)/Cl(-) exchange transporter 3	CLCN3	(17, 22)	(34)
IL-6 receptor	IL6R	(31)	(75)
JAM-A	F11R	(76)	(77)
Leucine-rich repeat-containing protein 4B	LRRC4B	(78)	(34)
Major prion protein (PrP^C^)	PRNP	(31, 79, 80)	(81, 82)
Plexin-B2	PLXNB2	(17, 22)	(34)
Protein tyrosine phosphatase receptor type F	PTPRF	(31)	(34)
Protocadherin-9	PCDH9	(31)	(34)
RAGE	RAGE	(83)	(84)
RANKL	TNFSF11	(85, 86)	(87)
Receptor-type tyrosine-protein phosphatase gamma	PTPRG	(17, 22, 31)	(34)
Receptor-type tyrosine-protein phosphatase kappa	PTPRK	(31)	(34, 88)
Semaphorin 4B	SEMA4B	(31)	(34)
Semaphorin 4D	SEMA4D	(31, 89)	(90)
Semaphorin-7A	SEMA7A	(17, 31)	(34)
SLAMF5^*^	CD84	(31)	(91)
UL16 binding protein 2	ULBP2	(92)	(93, 94)
VEGFR2	KDR	(95)	(96)
Vesicular integral-membrane protein VIP36	LMAN2	(17, 22)	(34)

* *confirmed pADAM10 substrates*.

### Canonical Shedding Activity of pADAM10

**Figure 1:**
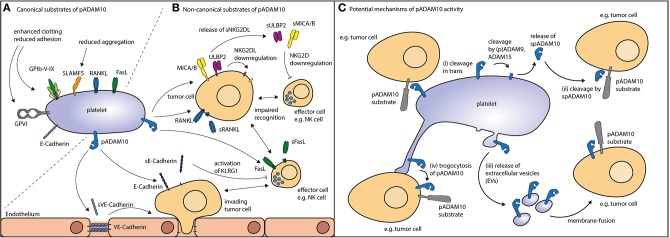
Potential routes of action for pADAM10. **(A)** Canonical substrates of pADAM10 (see Table 1) not only comprise molecular players involved in platelet function as exemplified by glycoproteins and SLAMF5 but also a plethora of immuoregulatory factors alike FasL and RANKL. **(B)** Non-canonical substrates of pADAM10 on other cells have been suggested to comprise NKG2DL MICA/B, ULBP2, and ectodomain shedding thereof reportedly dampens NK antitumor reactivity. Proteolytic cleavage of E-Cadherin (resulting in soluble E-Cadherin, sE-Cadherin) may inhibit NK effector functions. Cleavage of VE-Cadherin by pADAM10 may interfere with endothelial junctions thereby facilitating tumor invasion. Moreover, immunomodulation may be mediated by pADAM10 shedding of FasL from the surface of immune effector cells or shedding or RANKL e.g., from the surface of tumor cells. **(C)** Thus, pADAM10 may besides exerting proteolytic activity against substrates within the same membrane (*cis*), mediate (i) cleavage in *trans* (in the membrane of other cellular compounds). Alternatively, pADAM10 may be released as (ii) soluble form (spADAM10), (iii) contained in extracellular vesicles, or (iv) integrate in the membrane of the substrate-expressing cell.

pADAM10's classical mode of action (canonical shedding) is proteolytic cleavage of platelet-expressed substrates by membrane-anchored pADAM10. As of yet, established, i.e., experimentally confirmed, substrates of pADAM10 substrates in platelets primarily comprise molecules relevant for hemostasis (Figure 1A). ADAM10 is the main sheddase of the glycoproteins (GP)V (73) and GPVI (73, 97). GPVI is the collagen receptor of platelets and responsible for collagen-induced platelet activation (98), while GPV is part of the GPIb-IX-V complex, which is responsible for platelet activation upon encounter of vWF or upon binding of P-Selectin or αMβ2 expressed on endothelial cells or on neutrophils, respectively (99). ADAM10 activity is thus regulating key functions of platelet biology, although the role of shedded GP-fragments is not clear, neither under physiological conditions nor in disease (30). Soluble GPVI fragments can be detected in the blood (100) and a synthetic soluble GPVI fragment inhibited *in vitro* and *in vivo* platelet activation upon exposure to collagen (101). Of note, GPVI has been shown to promote metastasis in a mouse model (102) and it would thus be interesting to investigate how ADAM10 affects metastasis. Another confirmed substrate of pADAM10 is signaling lymphocyte activation molecule family member 5 (SLAMF5)/CD84, the exact function of which in platelet biology is currently unknown (91). No function of soluble SLAMF5/CD84 has been detected yet. SLAMF5/CD84 displays homophilic interaction and signals via EAT-2 and SAP (103). SLAMF5/CD84 in platelets seems to promote platelet aggregation by homophilic interaction (104), but *Slamf5/Cd84*^−/−^ mice did not display any phenotype regarding *in vitro* platelet activation and/or impaired hemostasis *in vivo* (105). Moreover, platelets express other known ADAM10 substrates which are not yet experimentally confirmed to be shed by pADAM10. These comprise molecules involved in signaling (PTPRF, PTPRK, PTPRG), adhesion (E-cadherin, Cadherin 6/10, CD44) or immunomodulation (CD40L, IL-6 receptor, RANKL, FasL, ULBP2) (see Table 1).

### Non-canonical Shedding Activity of pADAM10

pADAM10 may also exert proteolytic activity beyond the canonical shedding, i.e., shedding of platelet-derived substrates by membrane bound pADAM10 which we here summarize as non-canonical shedding of pADAM10. Interestingly, ADAM10 itself is subject to shedding by ADAM9 and ADAM15 (106, 107). Platelets express ADAM9, and analysis of the platelet sheddome has detected soluble pADAM10 (spADAM10) thereby further corroborating these results (31). Whether the soluble form of ADAM10 is proteolytically active is still a matter of debate (108), but clearly opens up a new channel for effects of pADAM10. Analysis of the degradome of murine soluble ADAM10 has been performed and suggested that soluble ADAM10 is in fact biological active (109). Another potential route of action might be cleavage of ADAM substrates in *trans*, this is when substrate and enzyme are located on different cells. While this is unusual for ADAM-proteases such effects have been described for ADAM10 in the context of Ephrin cleavage (110). In addition, platelets alike leukocytes and tumor cells can release extracellular vesicles (EVs), including exosomes and microvesicles/microparticles, which may be derived from resting as well as activated platelets (111, 112). Interestingly, the main source of EVs found in the plasma are in fact platelets and megakaryocytes (113, 114). Platelet-EVs are increasingly recognized as modulators of immunologic processes (115, 116). In the context of rheumatoid arthritis, platelet-EVs have been found to increase joint inflammation by eliciting an immune response from synovial fibroblasts (115). In addition, recent evidence indicates that platelet-EVs shape the function of regulatory T cells upon P-selectin dependent binding by yet not fully defined mechanisms (116). Of note, ADAM10 may reportedly be active in EVs, thereby being able to exert protease activity (117). Thus, it is tempting to speculate, that platelet-EVs carry active pADAM10 and act on cells other than platelets themselves. Another means to exert shedding on other cells could be facilitated by integration of pADAM10 into the membrane of cellular compounds, e.g., tumor cells (trogocytosis), which we could demonstrate to occur after tumor cell–platelet interaction, albeit not specifically for pADAM10 (7).

In the context of tumor-immunosurveillance, pADAM10 has not only been identified as sheddase of canonical substrates on platelets, but also to be involved in cleavage of the stress-induced NKG2D ligands (NKG2DL) MICA, MICB and ULBP2 from the surface of tumor cells (118, 119) (Figure 1B). Expression of NKG2DL is sensed by cytotoxic lymphocytes such as NK cells that express the cognate activating immunoreceptor NKG2D. Shedding of NKG2DL by ADAM10 (and/or ADAM17) in turn impairs tumor-cell lysis by NK cells. We recently observed reduced surface expression of NKG2DL on tumor cells upon coculture with platelets or exposure to platelet releasate (120). This was mirrored by enhanced detection of soluble NKG2DL, pointing to increased shedding of the surface expressed ligands. These observations were also confirmed by another group evaluating different tumor entities (121). Alike Cluxton and coworkers, we did not find enhanced mRNA-levels of tumor-*ADAM10* (or *ADAM17*) upon platelet coating. We thus concluded that pADAM10 (or pADAM17) might be responsible for the enhanced shedding and may act via one (or more) of the suggested non-canonical shedding pathways: (i) cleavage exerted in *trans* or (ii) cleavage mediated by soluble (s)pADAM10, (iii) conferred by platelet-derived EVs, or (iv) integration of pADAM10 in the substrate-bearing cell (trogocytosis).

Other interesting non-canonical pADAM10 substrates could be ligands for the epidermal growth factor receptor (EGFR) (122). There are seven well-established EGFR ligands, which are also expressed on tumor cells, and release of their ectodomains via shedding supports tumor progression in an auto/paracrine manner (123). Two (EGF and betacellulin) of the seven EGFR ligands are established substrates of ADAM10 (69) and shedding thereof could thus enhance existing EGFR signaling. Interestingly, EGF itself is expressed on platelets (Table 1) and its release by tumor-coating platelets was shown to support key features of tumor progression (67). In this study, however, release of platelet-derived EGF was shown to depend mainly on platelet derived ADAMDEC-1 rather than pADAM10 (67, 124).

The chemokine CX3CL1 (fractalkine) constitutes another putative target of pADAM10 and exists either as a membrane-anchored form or is released via ectodomain shedding mediated by ADAM10 (125). CX3CL1 is mainly expressed on endothelial cells (126) but can also be found on tumor cell lines of several entities (127, 128). Its cognate receptor CX3CR1 is expressed on NK cells and T cells (129) but also on tumor cells (130). Of note, platelets express CX3CR1 binding of which was shown to mediate platelet activation and enhance adhesion to collagen and fibrinogen (131). In the context of NK cell or T cell mediated immunosurveillance of cancer cells, signaling via the CX3CR1/CX3CL1 axis might either aid or impair cancer progression [reviewed in (129)]. How pADAM10 could modulate the CX3CR1/CX3CL1 axis in this context has not yet been investigated.

Despite putative shedding of EGFR ligands or chemokines, immunomodulation via the above mentioned non-canonical modes of action may as well be executed by pADAM10 on several other substrates alike FasL, RANKL, E-Cadherin, VE-Cadherin (70, 71, 85, 132, 133) (Figure 1C).

### Platelet Mediated Activation/Inhibition of (p)ADAM10

The activity of (p)ADAM10 is highly regulated on multiple levels including transcription, translation and posttranslation [reviewed in (108, 134, 135)] Once translated, full-length ADAM10 trafficks through the secretory pathway where it is activated by removal of its prodomain (136). Substrate-access to the catalytic site of mature ADAM10 is still tightly regulated through conformational changes involving its cysteine-rich domain (137). The process of ADAM10 trafficking/maturation is closely connected to the Tetraspanin (Tspan)C8 group (30). This group of six transmembrane proteins includes Tspan5, 10, 14, 15, 17, and 33 which all have been shown to associate with ADAM10, thereby promoting its trafficking to the cell surface as well as its maturation (138). Accumulating evidence indicates that ADAM10 maintains its association with TspanC8 members and that different TspanC8 partners may guide ADAM10-activity toward different substrates suggesting also interesting therapeutic implications (30). The TspanC8 members 14, 15, and 33 have also been identified in human platelets (139, 140) and Tspan33 is among the 40 most frequent platelet-membrane proteins (22). Platelet-expressed TspanC8 members might therefore play an important role in pADAM10 shedding events (30). As yet only indirect evidence, based on transfectants, is available suggesting Tspan14 as a negative regulator of GPVI shedding (140). ADAM10 activity might additionally be modulated by the dynamic composition of the lipid-bilayer by mechanisms such as trapping of substrates within cholesterin-rich lipid-rafts which are usually devoid of ADAM10 [reviewed in (141)]. Moreover, Ca^2+^ and calmodulin are important regulators of ADAM10 activity (142) also in platelets (135). It was suggested that pro-ADAM10 associates with calmodulin and that Ca^2+^ influx allows ADAM10-maturation by disrupting this association (56). Likewise, in platelets, Ca^2+^ influx has been shown to rapidly and potently increase pADAM10 reactivity (74). Of note, calmodulin was also shown to associate with substrates of pADAM10 such as GPVI on platelets thereby putatively negatively regulating their proteolysis (73, 143). Increase of intracellular Ca^2+^ thus represents a mechanism to rapidly regulate (p)ADAM10-mediated shedding events although the exact underlying processes are not yet fully elucidated.

However, it should be considered that platelet-derived factors could also influence the ADAM10 activity in the platelet-microenvironment. TGFβ has been shown to enhance transcription of *Adam10* (and *Adam17*) in renal cells (144), and platelets can release large amounts of active TGFβ (145). However, neither Cluxton nor our group detected enhanced *ADAM10* (or *ADAM17*) transcription in tumor cells upon exposure to platelets (or platelet releasate). This suggests that specific mechanisms govern transcription of *ADAM10* in distinct cell-types. Moreover, it has been shown that thrombin activates PAR-1 receptors on endothelial cells, leading to increased activity of ADAM10 which enhanced cleavage of VE-cadherin leading to facilitation of T cell transmigration (133). Similarly, one might speculate that thrombin released by platelet-covered tumor cells might also facilitate transmigration of tumor cells during metastasis. It should be noted that tumor cells can also express PAR-1, and overexpression of PAR-1 has been implicated in enhanced metastasis (146, 147). PAR-1 signaling in tumor cells has been shown to enhance metastasis in experimental models (148), which is attributed to initiation of an EMT-program (149). The source of thrombin, the main activator of PAR-1, was suggested to be the tumor cells themselves (148), but, in the context of metastasizing tumor cells, coating platelets might additionally contribute to thrombin generation (150). Since ADAM10 is also activated by thrombin as described above (133) and has been implicated in enhanced tumor cell invasiveness (151), it appears possible that in fact activated platelets contribute to tumor cell invasiveness by regulation of ADAM10 via thrombin/PAR-1 signaling (138). ADAM10 is inhibited by TIMP-1 and TIMP-3, with TIMP-1 being inhibitory at lower concentration compared to TIMP-3 (53). Platelets contain, among other TIMPs, TIMP-1, and TIMP-3, which are independently stored in distinct platelet compartments, but are rapidly and completely released upon activation with thrombin (152). Another physiological inhibitor of ADAM10 is the membrane-anchored RECK (153) and has been detected in the platelet membrane proteome (22). The reason for the presence of both ADAM10 and its inhibitors in platelets remains to be thoroughly elucidated and suggests complex context-specific regulation of protease-activity.

## Conclusion and Perspective

Platelets are associated with pathophysiology and may influence efficacy of systemic treatment in several diseases. It is thus an appealing idea to take advantage of platelet-derived biomarkers for lipid biopsies (113), especially since platelets may display a malignant phenotype (154). In line, both platelets and platelet-derived EVs have been suggested as putative biomarker (155, 156). The soluble form of ADAM10 may still exert proteolytic activity, however it remains unclear under which instances or toward which targets (108). The potential therapeutic targeting of ADAM10 activity is, while promising in many instances, likely to result also in side effects, given its variety of biological substrates. The closely related ADAM17 which is also expressed on platelets may exert similar activity, and targeting ADAM10 may thus also affect cleavage by ADAM17, especially since (i) hypothetically specific inhibitors still show cross-inhibition of ADAM17 and (ii) ADAM17 may compensate for a lack of ADAM10 shedding events (157). Since distinct tetraspanins are thought to guide ADAM10 substrate shedding, targeting of a specific tetraspanin-ADAM10 complex may thus constitute a promising approach to modulate ADAM10 proteolytic activity toward defined substrates. Further studies unraveling the regulation of ADAM10 activity, also with regard to protease localization (membrane bound, EV, or as soluble truncated form) and shedding of specific substrates are certainly warranted. ADAM10 exerts protease activity on a plethora of factors relevant beyond hemostasis. With this review, we aimed to provide a timely overview about the knowledge of (p)ADAM10, its substrates on platelets and on their microenvironment.

## Author Contributions

All authors listed have made a substantial, direct and intellectual contribution to the work, and approved it for publication. Conceptualization: H-GK, HS, KK, and SM. Writing-original draft preparation: KK and SM. Writing-review and editing: H-GK and HS. Visualization: KK.

### Conflict of Interest

The authors declare that the research was conducted in the absence of any commercial or financial relationships that could be construed as a potential conflict of interest.
